# The diversity of CO_2_-concentrating mechanisms in marine diatoms as inferred from their genetic content

**DOI:** 10.1093/jxb/erx163

**Published:** 2017-05-16

**Authors:** Chen Shen, Christopher L Dupont, Brian M Hopkinson

**Affiliations:** 1 Qingdao Institute of BioEnergy and BioProcess Technology, Chinese Academy of Sciences, Qingdao, China; 2 Department of Marine Sciences, University of Georgia, Athens, GA, USA; 3 J. Craig Venter Institute, La Jolla, CA, USA

**Keywords:** Carbon dioxide, carbonic anhydrase, diatom, marine, photosynthesis, transporters

## Abstract

Marine diatoms are one of the most ecologically significant primary producers in the ocean. Most diatoms use a CO_2_-concentrating mechanism (CCM) to overcome the scarcity of CO_2_ in the ocean and limitations of the carbon-fixing enzyme Rubisco. However, the CCMs in model diatoms differ substantially in their genetic make-up and structural organization. To assess the extent of CCM diversity in marine diatoms more generally, we analyzed genome and transcriptome data from 31 diatom strains to identify putative CCM genes, examine the overall CCM architecture, and study CCM development in the context of the evolutionary history of these diatoms. Key CCM genes [carbonic anhydrases (CAs) and solute carrier 4 (SLC4) bicarbonate transporters] identified in the diatoms were placed into groups of likely orthologs by sequence similarity (OrthoMCL) and phylogenetic methods. These analyses indicated that diatoms seem to share similar HCO_3_^−^ transporters, but possess a variety of CAs that have either undergone extensive diversification within the diatom lineage or have been acquired through horizontal gene transfer. Hierarchical clustering of the diatom species based on their CCM gene content suggests that CCM development is largely congruent with evolution of diatom species, despite some notable differences in CCM genes even among closely related species.

## Teaser

Analysis of key CO_2_-concentrating mechanism components in the transcriptomes and genomes of marine diatoms shows that the system is diverse and this diversity largely follows phylogenetic lines.

## Introduction

Diatoms, a group of unicellular photoautotrophic algae, are one of the most ecologically significant primary producers in the ocean (Tréguer *et al.*, 1995; Field *et al.*, 1998; Falkowski *et al.*, 2000). Diatoms can actively take up both CO_2_ and HCO_3_^−^ for photosynthesis, and this ability to take up dissolved inorganic carbon (DIC) rapidly is critical to their high primary productivity (Matsuda *et al.*, 2001; Trimborn *et al.*, 2008). Dissolved CO_2_ availability is limited in the ocean due to the high pH of seawater and its slow diffusion rate in water compared with air (Goyet and Poisson, 1989; Raven, 1994; Matsuda *et al.*, 2001). Moreover, the concentration of CO_2_ (~10–25 µM) in modern seawater is not sufficient to saturate rates of carbon fixation by Rubisco, the principal enzyme that catalyzes carbon fixation in the Calvin–Benson cycle (Badger *et al.*, 1998). To overcome these difficulties, diatoms, as well as many other marine phytoplankton, developed systems called CO_2_-concentrating mechanisms (CCMs) to actively take up inorganic carbon (C_i_) and increase the CO_2_ concentration around Rubisco (Colman and Rotatore, 1995; Burkhardt *et al.*, 2001; Matsuda *et al.*, 2001; Rost *et al.*, 2003).

Most diatoms use some variety of biophysical CCM, which depends on active pumping of C_i_ across cellular membranes (Roberts *et al.*, 2007; Trimborn *et al.*, 2009). Generally, biophysical CCMs consist of CO_2_ and HCO_3_^−^ transport mechanisms, intra- and extracellular carbonic anhydrases (CAs), enzymes that catalyze the reversible dehydration of HCO_3_^−^ to CO_2_, and a microcompartment in which Rubisco is concentrated (the pyrenoid in eukaryotes), which helps minimize the diffusive leakage of CO_2_. However, some diatom species such as *Thalassiosira weissflogii* may use a biochemical C_4_ mechanism, which employs a four-carbon organic intermediate for internal translocation, though the C_4_ pathway’s existence and importance remain controversial (Reinfelder *et al.*, 2000; Roberts *et al.*, 2007).

Diatoms have a complicated evolutionary history including multiple endosymbiotic events, which involved unknown host cells as well as cyanobacteria, a red alga, and possibly a green alga (Kroth, 2002; Moustafa *et al.*, 2009). As a result of a secondary endosymbiotic event, a complicated four-layer chloroplast membrane system was formed (Gibbs, 1981). This compartmentalization makes diatom CCMs more complex since the location of CAs and HCO_3_^−^ transporters can vary among different diatom species (Kroth *et al.*, 2008; Samukawa *et al.*, 2014). There is still no consensus about the ultimate origin of CCMs, but the CCM components in prokaryotes and eukaryotes show little to no homology, suggesting that they evolved independently. Furthermore, it is likely that CCMs evolved independently in the major eukaryotic algal lines (Badger *et al.*, 1998, 2002; Raven *et al.*, 2011, 2012). Young *et al.* (2012) have shown that form ID Rubisco in Bacillariophyta (diatoms) and Haptophyta underwent positive selection during low-CO_2_ episodes in geological history, which possibly relates to the origin of CCMs in these groups.

CCMs in marine diatoms have been most well studied in the model diatoms *Phaeodactylum tricornutum* and *Thalassiosira pseudonana* (Tachibana *et al.*, 2011; Samukawa *et al.*, 2014; Hopkinson *et al.*, 2016). Ten putative HCO_3_^−^ transporters from solute carrier 4 (SLC4) and solute carrier 26 (SLC26) protein families have been found in the *P. tricornutum* genome, and one of these SLC4 genes has been functionally characterized to encode a HCO_3_^−^ transporter (Nakajima *et al.*, 2013). SLC4 homologs also exist in *T. pseudonana* (Nakajima *et al.*, 2013), and though these homologs have not been functionally characterized, the SLC4 family has a narrow substrate specificity, with nearly all characterized members transporting bicarbonate, though counter ions transported with HCO_3_^−^ differ (Romero *et al.* 2004; Parker and Boron, 2013). No homologs to HCO_3_^−^ transporters from other gene families, such as SbtA from cyanobacteria and LCI1 from the green alga *Chlamydomonas reinhardtii*, have been found in diatom genomes, suggesting that SLC family transporters are the primary mechanism for HCO_3_^−^ transport in diatoms.

There is some overlap in the families of CAs present in the two species. Both species contain α- and γ-CAs, but β-CAs have been found only in *P. tricornutum* and δ-CAs and ζ-CAs have been found only in *T. pseudonana* (Tachibana *et al.*, 2011; Samukawa *et al.*, 2014). The spatial distribution of CAs differs dramatically in the two diatoms. In *T. pseudonana*, CAs are present in the periplasmic space, serving to convert HCO_3_^−^ to CO_2_ for uptake, whereas no such CAs are found in *P. tricornutum*. Additionally, a CA is localized to the cytoplasm of *T. pseudonana*, which probably serves to convert CO_2_ diffusing into the cell to HCO_3_^−^, but *P. tricornutum* lacks cytoplasmic CA. Instead, *P. tricornutum* has multiple CAs within the four-layered chloroplast membrane, which probably serve to convert both CO_2_ diffusing into the cell and CO_2_ leaking out of the chloroplast into HCO_3_^−^. *Thalassiosira pseudonana* has only one CA within the chloroplast membrane subcompartments. In the chloroplast, *P. tricornutum* lacks CAs in the bulk stroma, allowing a HCO_3_^−^ pool to accumulate, but this diatom has two CAs in the pyrenoid, where Rubisco is localized, serving to convert the accumulated HCO_3_^−^ pool to CO_2_. In contrast, *T. pseudonana* has a CA distributed throughout the chloroplast stroma, which would be expected to complicate subcellular concentration of a HCO_3_^−^ pool, suggesting that the CCM works quite differently in this diatom (Samukawa *et al.*, 2014). Very recently, Kikutani *et al.* (2016) have identified a new class of diatom CA, θ-CAs, which was first characterized in *P. tricornutum* where its presence in the pyrenoid-penetrating thylakoid suggests that it is involved in generating CO_2_ for Rubisco. A homolog is present in *T. pseudonana* and may function similarly in this species.

This diversity of CCMs in diatoms is not necessarily unexpected as the group is evolutionarily diverse and subject to extensive horizontal gene transfer. Diatoms emerged ~180 Mya (Medlin, 2016) and have subsequently evolved into a number of lineages that are found in diverse aquatic environments (the ocean, lakes, rivers, etc.). Sequencing of several diatom genomes has revealed complex and diverse genomes with significant contributions from the ancestral algal endosymbiont, bacteria, and potentially other eukaryotes, in addition to the core genome from the ancestral eukaryotic host (Armbrust *et al.*, 2004; Bowler *et al.*, 2008; Mock *et al.*, 2017).

The dramatic differences in the CCM components and architecture of two model diatoms and new availability of diatom genomic or transcriptomic data sets motivated us to explore CCM diversity in diatoms more generally. In this work, we examine the CCM gene content of diverse diatoms based on analyses of the four available genome sequences and 30 diatom transcriptomes from the Marine Microbial Eukaryote Transcriptome Sequencing Project (MMETSP; Keeling *et al.*, 2014; Table 1). We focus on genes that would be involved in both biophysical and biochemical CCMs, namely CAs and SLC4 bicarbonate transporters. Genes involved specifically in C_4_ metabolism are not examined since the pathway appears to be rare among diatoms (Reinfelder, 2011; Hopkinson *et al.*, 2016). Furthermore, while the genes involved in the C_4_ pathway are ubiquitously distributed, a functioning C_4_ pathway depends critically on the localization and expression level of the proteins involved, information that is not fully available in the genomes and transcriptomes. After grouping CCM component genes into families of putative orthologs, the CCM gene content of the diatoms are compared with each other using hierarchical clustering, and finally the similarity of CCMs among species (based on the hierarchical clustering) is compared with the evolutionary history of diatoms.

**Table 1.** Diatom strains analyzed in this study, their morphological group, the source of the data set, and total number of peptide sequences in the genome or transcriptome data set

Species	Morphology	Genome/transcriptome	Peptides
*Amphora coffeaeformis* CCMP127	Raphid pennate	Transcriptome	13 596
*Amphiprora* sp.	Raphid pennate	Transcriptome	18 334
*Fragilariopsis kerguelensis* L2_C3	Raphid pennate	Transcriptome	33 049
*Fragilariopsis cylindrus* CCMP1102	Raphid pennate	Genome	45 214
*Nitzschia punctata* CCMP561	Raphid pennate	Transcriptome	17 709
*Pseudo-nitzschia fraudulenta* WWA7	Raphid pennate	Transcriptome	41 247
*Pseudo-nitzschia australis* 10249_10_AB	Raphid pennate	Transcriptome	15 156
*Pseudo-nitzschia multiseries* CLN-47	Raphid pennate	Genome	19 703
*Phaeodactylum tricornutum*	Raphid pennate	Genome	10 402
*Thalassiothrix antarctica* L6_D1	Araphid pennate	Transcriptome	18 200
*Thalassionema nitzschioides* L26_B	Araphid pennate	Transcriptome	16 133
*Chaetoceros affinis* CCMP159	Polar centric	Transcriptome	14 260
*Chaetoceros debilis* MM31A_1	Polar centric	Transcriptome	15 052
*Chaetoceros neogracile* CCMP1317	Polar centric	Transcriptome	18 670
*Ditylum brightwellii* GSO103	Polar centric	Transcriptome	17 187
*Ditylum brightwellii* GSO104	Polar centric	Transcriptome	21 584
*Ditylum brightwellii* GSO105	Polar centric	Transcriptome	17 773
*Extubocellulus spinifer* CCMP396	Polar centric	Transcriptome	43 366
*Corethron pennatum* L29A3	Radial centric	Transcriptome	39 296
*Proboscia alata* PI_D3	Radial centric	Transcriptome	25 076
*Skeletonema dohrnii* SkelB	Radial centric	Transcriptome	19 615
*Skeletonema marinoi* SkelA	Radial centric	Transcriptome	17 193
*Skeletonema menzelii* CCMP793	Radial centric	Transcriptome	13 490
*Thalassiosira antarctica* CCMP982	Radial centric	Transcriptome	24 242
*Thalassiosira gravida* GMp14c1	Radial centric	Transcriptome	17 050
*Thalassiosira oceanica* CCMP1005	Radial centric	Transcriptome	28 635
*Thalassiosira pseudonana* CCMP1335	Radial centric	Genome	11 776
*Thalassiosira rotula* CCMP3096	Radial centric	Transcriptome	22 123
*Thalassiosira rotula* GSO102	Radial centric	Transcriptome	19 160
*Thalassiosira weissflogii* CCMP1010	Radial centric	Transcriptome	15 593
*Thalassiosira weissflogii* CCMP1336	Radial centric	Transcriptome	14 286

## Materials and methods

### Data sets

Sets of protein sequences for 34 diatom strains were obtained from the four available genomes (*Thalassiosira pseudonana* CCMP1335; *Phaeodactylum tricornutum*; *Fragilariopsis cylindrus* CCMP1102; and *Pseudo-nitzschia multiseries* CLN-47) and from 30 diatom transcriptomes sequenced by the MMETSP (Keeling *et al.*, 2014). Genomes were obtained from the JGI website (Nordberg *et al.*, 2014), and transcriptome data were obtained from the iMicrobe website. Based on examinations of putative completeness, assembly quality, and bacterial contamination, the following data sets were excluded: *Asterionellopsis glacialis* CCMP134, *Chaetocerous curvisetus*, and *Thalassiosira miniscula*. All three of these EST libraries were excluded as an LPI (‘lineage probability index’) analysis (Podell and Gaasterland, 2007) showed that <90% of the predicted peptides were of eukaryotic origin, which suggests biological contamination. The remaining ESTs and genomes have LPI predictions of >99% eukarya. After excluding these data sets, 4 genomes and 27 transcriptomes were further analyzed (Table 1).

These diatoms come from four major morphological groups: polar centrics (7 data sets), radial centrics (13 data sets), raphid pennates (9 data sets), and araphid pennates (2 data sets). Modern molecular phylogenetic analyses have revealed that these morphological groups have meaningful correspondences with phylogeny, but not all the morphologically defined groups form monophyletic clades (Medlin *et al.*, 1996; Kooistra *et al.*, 2003). In this work, the morphological designations are used since these distinctions are convenient for discussing relationships between CCM genetic structure and diatom phylogeny. The centric diatoms have roughly radial symmetry while pennates are elongated along one axis. Radial centrics are mainly circular in outline while the bi- or multipolar diatoms (polar centrics) have non-circular outlines, including triangular and quadrangular forms. Pennates are divided into the raphid pennates, distinguished by a pair of slits running longitudinally along the frustule (the raphe) that is used in motility, and araphid pennates that lack such structures.

### Identification of putative CCM genes in diatom protein sequences

Query sequence sets of SLC4 bicarbonate transporters and CAs (α-CAs, γ-CAs, and δ-CAs) were collated from the four genomes based on published work (Tachibana *et al.*, 2011; Nakajima *et al.*, 2013; Samukawa *et al.*, 2014) and gene annotation in JGI (Armbrust *et al.*, 2004; Bowler *et al.*, 2008). The query data sets were manually curated and then used to BLAST against the database of diatom protein sequences with a stringent e-value cut-off of 10^–5^. Hidden Markov Model (HMM) analyses (Eddy, 1998) were also used to identify possible CAs and HCO_3_^−^ transporters using an e-value cut-off of 10^–5^. The HMMs were constructed from the same sets of sequences used in the BLAST queries. Sequences that scored below the 10^–5^ cut-off in both the BLAST and HMM analyses were retained. Sequences were aligned using ClustalX 2.1 (Larkin *et al.*, 2007), and the alignments were examined to identify gene fragments and duplicates, which were removed manually. The average length (±SD) of complete peptides for these data sets were: α-CAs, 447 ± 180; γ-CAs, 242 ± 73; δ-CAs, 300 ± 41; and SLC4 bicarbonate transporters, 568 ± 145 amino acids. Searches for β- and ζ-CAs identified sequences in only a few diatoms, and so these families were not investigated further.

### Grouping of diatom CCM genes

The putative CA (α-CAs, δ-CAs, and γ-CAs) and SLC4 bicarbonate transporter protein sequences identified in the diatoms were grouped into sets using two approaches: (i) OrthoMCL, a program that uses sequence similarity to identify likely orthologs; and (ii) a phylogenetic approach using maximum parsimony trees.

OrthoMCL (Chen *et al.*, 2006) was used to cluster components of SLC4 bicarbonate transporters, α-CAs, δ-CAs, and γ-CAs into groups that represent putative orthologs and ‘recent’ paralogs. Default parameters were used throughout most of the analysis, and in the final clustering step the default value (1.5) was used for the inflation parameter for SLC4 bicarbonate transporters, α-CAs, and γ-CAs components, but the inflation parameter was set to 4 for δ-CAs, which otherwise formed only three or four groups.

A phylogenetic approach was also taken for comparison with the OrthoMCL results. Sequences from each CCM component were aligned by ClustalX 2.1 (Larkin *et al.*, 2007) and the alignments were trimmed using the Gblocks Server (Talavera and Castresana, 2007) and adjusted manually. Maximum parsimony trees were then built using MEGA 6.06 (Tamura *et al.*, 2013), and a bootstrap analysis using 100 resamplings was conducted. The general approach used to define sequence groups was to start from the leaves of the tree and identify ever larger clades of sequences with >50% bootstrap support. Enlargement of a group was stopped when bootstrap support for more ancestral nodes fell below 50%. Groups with fewer than four sequences were ignored. This approach was used to define most protein sequence groups. However, in some cases, groups were expanded past an internal node whose support fell below 50% when it was deemed to lead to more reasonable groups. Typically, this occurred when the poorly supported internal node defined the position of a single sequence relative to a larger clade of sequences. In rare cases, two small clades of sequences from phylogenetically similar species were combined despite the node linking the two clades falling below 50% bootstrap support. These criteria were developed to ensure the groupings had reasonable phylogenetic support and that the major groups contained a substantial number of sequences for comparison with the OrthoMCL groups.

### Hierarchical clustering of the diatom species based on CCM gene content

Matrices were compiled in which each row represents a diatom species and each column represents a CCM gene group defined using either OrthoMCL or phylogenetic methods. The values of each entry in the matrix represent the number of proteins from a CCM gene group found in the diatom species. For consistency with the phylogenetic groups, OrthoMCL groups with <4 sequences among all the diatoms were removed. The two matrices, one based on the OrthoMCL groups and the second based on the phylogenetic groups, were used in a hierarchical clustering analysis in R (hclust). A similarity matrix was formed based on Euclidean distances, and a dendrogram was constructed from the matrix using complete-linkage cluster analysis in R. Comparison of the two hierarchical trees was conducted in R using the package dendextend (Galili, 2015). Comparison of each hierarchical tree with the diatom 18S rDNA phylogenetic tree was conducted in R using the ape and phytools packages (Paradis *et al.*, 2004; Revell, 2012). As a statistical test of congruence between trees, the CADM (Congruence Among Distance Matrices) test was used with 999 random permutations of distance matrices (Campbell *et al.*, 2011).

### Diatom species phylogeny

A diatom phylogenetic tree was constructed based on the 18S rDNA of the 29 diatoms strains, with *Bolidomonas pacifica* L. Guillou & M.-J. Chretiennot-Dinet used as the outgroup (GenBank ID HQ912557.1). The 18S rDNA sequences were downloaded from the MMETSP website (http://marinemicroeukaryotes.org/resources-files/18s.fa) and GenBank. The 18S rDNA sequence of *Chaetoceros affinis* was not available. The sequences were aligned using the SILVA Incremental Aligner tool online (SINA, http://www.arb-silva.de/aligner/;Pruesse *et al.*, 2012), which is specifically designed to align rRNA gene sequences. The best nucleotide substation model was found and a maximum likelihood tree was built using Mega 6.06.

## Results

### Classification of CCM gene families using OrthoMCL

**Fig. 1.**
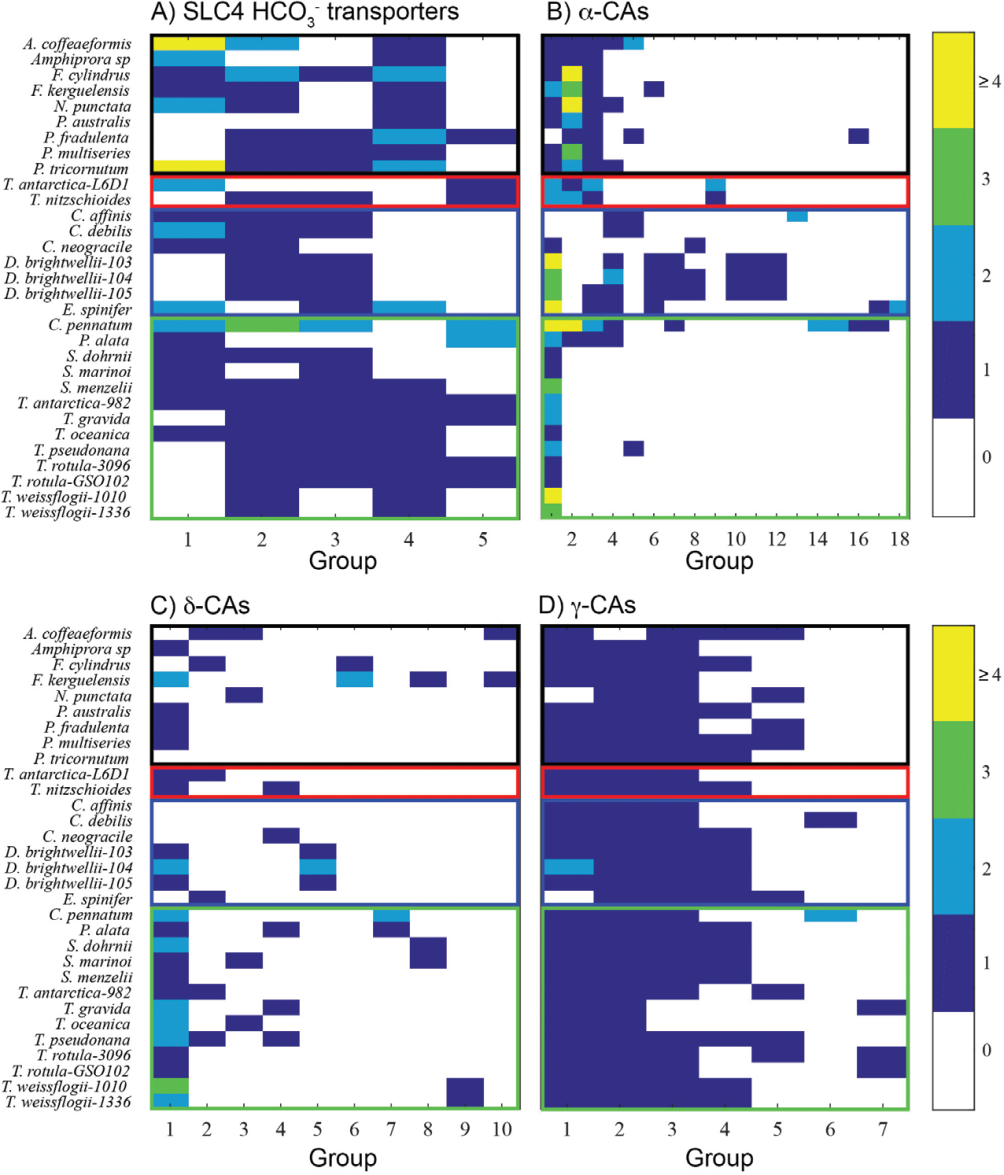
Results of OrthoMCL analysis of CCM genes in marine diatom species. (A) SLC4 bicarbonate transporters, (B) α-CAs, (C) δ-CAs, (D) γ-CAs. For each protein family, the different sequence groups are ordered from most to least abundant along the *x*-axis and the diatom strains are on the *y*-axis. The number of proteins of each group in each diatom strain is indicated by the color of the rectangle. The open rectangles outlining sets of species indicate different diatom morphological groups: black, raphid pennates; red, araphid pennates; blue, polar centrics; green, radial centrics.

Four CCM gene data sets (SLC4 bicarbonate transporters, α-CAs, δ-CAs, and γ-CAs) were compiled from the protein sequences of 31 diatom strains. β-CAs and ζ-CAs were not included because these genes were found in very few diatoms. OrthoMCL analysis was used to classify protein sequences from each family into 5–18 groups (Fig. 1). The SLC4 bicarbonate transporters were clustered into five groups, and most diatom species had representatives from three or four of the five groups, indicating that diatom species share a generally conserved set of SLC4 bicarbonate transporters (Fig. 1A), although there has been some diversification in certain species. For example, the three transporters from *T. pseudonana* fell into three different OrthoMCL groups, and the nine SLC4 bicarbonate transporters from *P. tricornutum* fell into four different OrthoMCL groups.

In contrast, CAs were generally split into a larger number of groups (7–18) and often the CAs within these OrthoMCL groups were derived from a single diatom genus or even a single diatom species, representing lineage-specific gene duplications. This trend was most notable in the α-CAs, which were split into 18 groups, with only one group having sequences from more than half of the diatom strains (Fig. 1B). δ-CAs were split into 10 groups, with one group containing δ-CAs from 23 strains and the remaining groups only contained sequences from ≤6 strains (Fig. 1C). Among the seven γ-CAs groups, three groups contained sequences from 29–30 strains, and most strains possessed one γ-CA in each of the three groups. One γ-CA group contained sequences from 18 strains, while the remaining groups contained sequences from <10 strains (Fig. 1D).

### Classification of CCM gene families using protein phylogenies

**Fig. 2.**
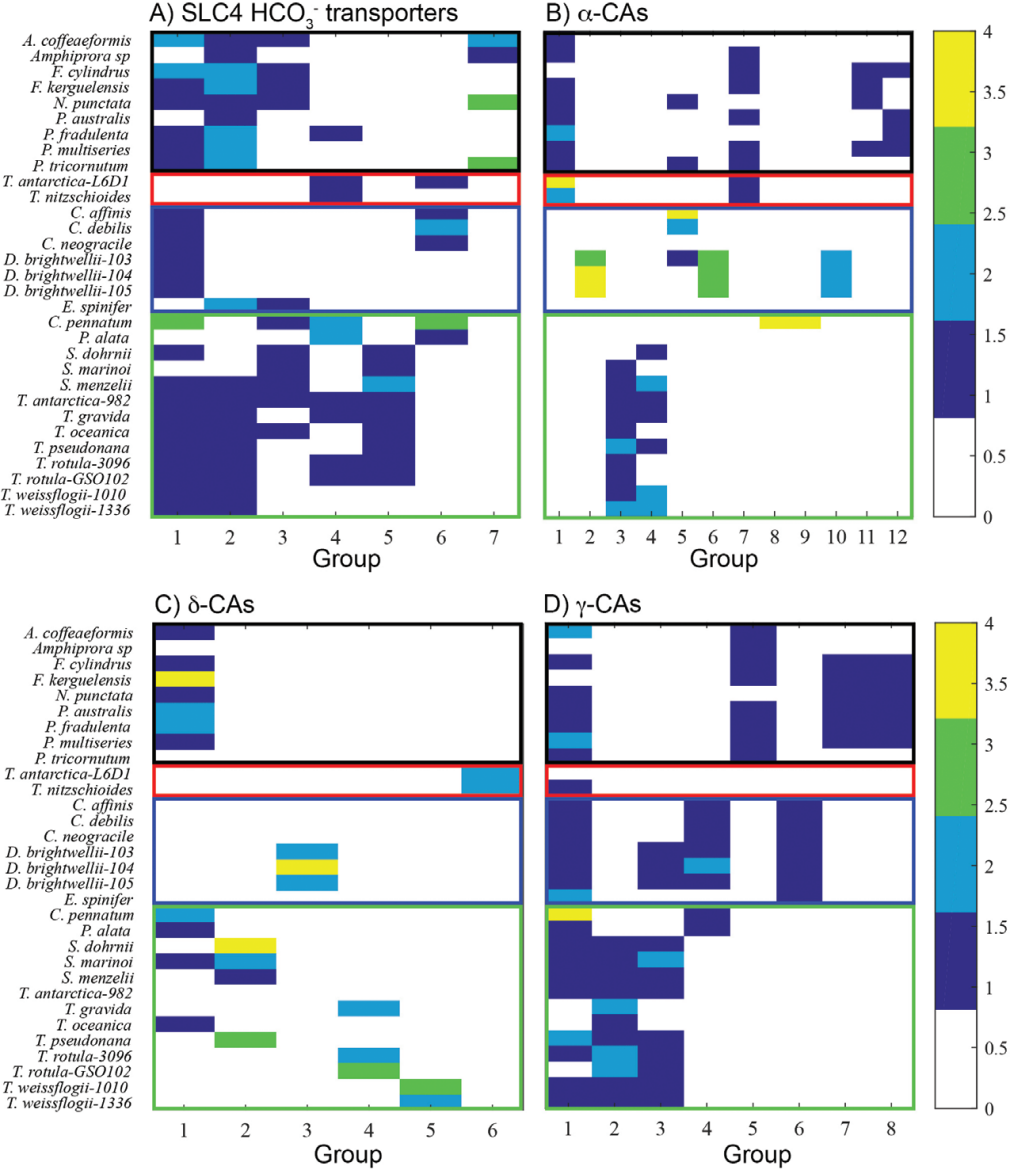
Results of a phylogenetic analysis of CCM genes in marine diatoms species. (A) SLC4 bicarbonate transporters, (B) α-CAs, (C) δ-CAs, (D) γ-CAs. For each protein family, the different sequence groups are ordered from most to least abundant along the *x*-axis and the diatom strains are on the *y*-axis. The number of proteins of each group in each diatom strain is indicated by the color of the rectangle. The open rectangles outlining sets of species indicate different diatom morphological groups: black, raphid pennates; red araphid pennates; blue, polar centrics; green, radial centrics.

**Fig. 3.**
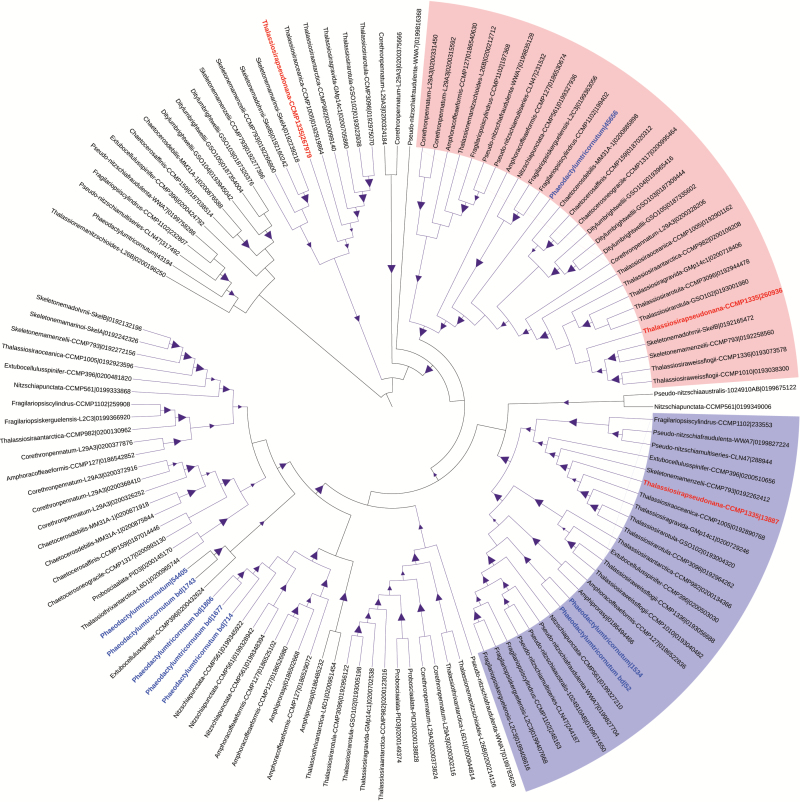
Maximum parsimony tree of putative SLC4 bicarbonate transporters. Triangular symbols indicate bootstrap value ≥50% and different colors indicate selected groups determined using the criteria described in the Materials and methods. The red text indicates transporters from *T. pseudonana* and blue text indicates sequences from *P. tricornutum*. The first two large groups contained sequences from most strains. Group 1 (red clade in the circular tree) has sequences from 29 strains and group 2 (purple clade in the circular tree) has sequences from 25 strains.

We also inferred phylogenies with the protein sequences of each CCM component and used maximal clades with >50% support at most nodes as a criterion for defining a group. Several different approaches to building phylogenetic trees were tried (including several methodologies to build maximum likelihood trees), but by far the most well-supported trees were generated using maximum parsimony methods. Even in this best case, a significant number of sequences were not placed into well-supported groups (>50% bootstrap support) or the groups were very small (<4 sequences). While a total of 460 sequences were classified using OrthoMCL, only 358 were successfully classified using phylogenetic methods. Nonetheless, general trends found were with the classifications obtained using OrthoMCL. SLC4 bicarbonate transporters were found in several large clusters, two of which included sequences from most strains, again indicating that diatoms share a set of similar SLC4 bicarbonate transporters (Figs 2, 3).

**Fig. 4.**
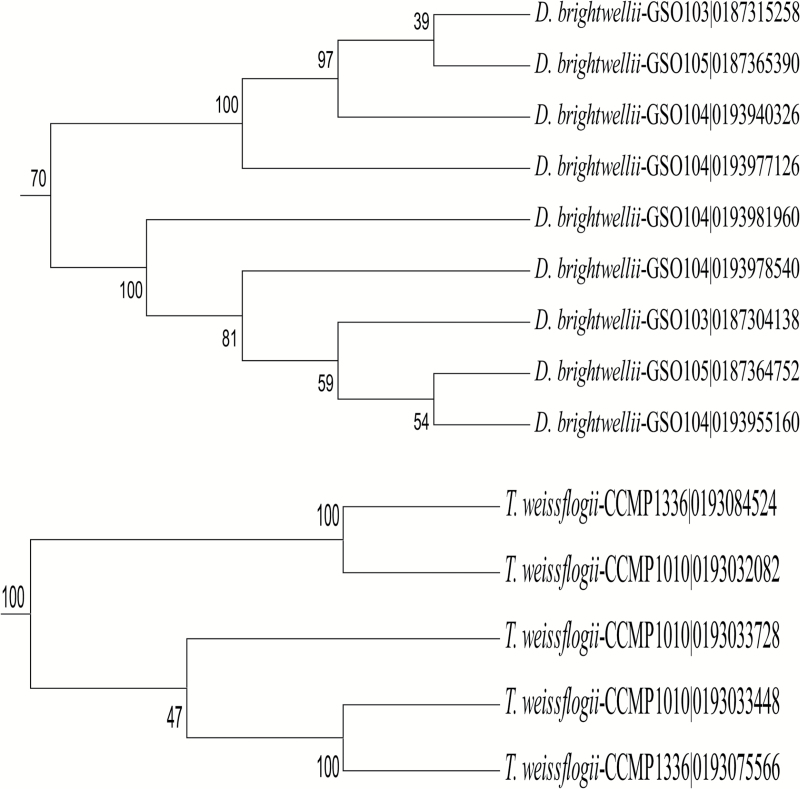
Subtrees from a maximum parsimony tree of δ-CAs, showing that identified groups typically are derived from a specific lineage.

On the other hand, phylogenetic trees of CAs tended to form groups within genera or species, much like what was observed in the OrthoMCL analysis. For example, in the δ-CAs there are several groups that are species specific, most notably a cluster of sequences from three *Ditylum brightwellii* strains (Figs 2, 4; Supplementary Fig. S1 at *JXB* online). α-CA and γ-CA trees also have similar groupings, as shown in Supplementary Figs S2 and S3. In groups defined by the phylogenetic method, CA sequences from araphid pennates and the radial centrics *Corethron pennatum* L29A3 and *Proboscia alata* PI_D3 often did not fall into the defined groups as a result of low bootstrap support, which suggests that their CAs are quite different from those of other diatoms.

### Hierarchical clustering of diatom species based on CCM gene content

**Fig. 5.**
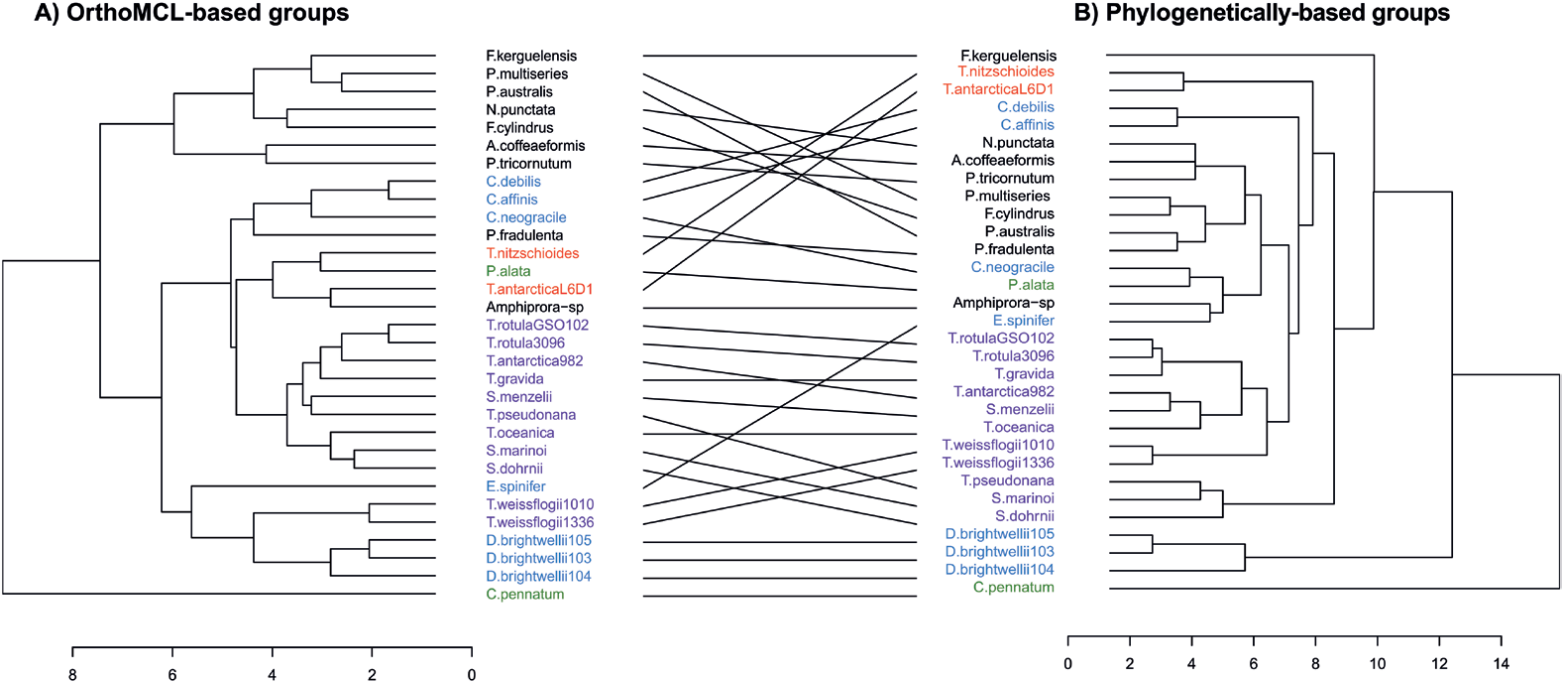
Comparison of two hierarchical clusterings of 31 diatom strains in terms of their CCM gene content as grouped by OrthoMCL and protein phylogeny. Black, raphid pennates; red, araphid pennates; blue, polar centrics; purple, radial centrics belonging to order Thalassiosirales; green, other radial centrics.

Two independent hierarchical clusterings of diatom species based on their genome-encoded CCM gene content were generated, the first using protein groupings identified with OrthoMCL and the second using protein groupings determined from protein phylogenies. Comparison of the two methods shows reasonable agreement, despite some notable exceptions such as the placement of three raphid pennates (*Amphiprora* sp., *Pseudo-nitzschia fraudulenta* WWA7, and *Fragilariopsis kerguelensis*) (Fig. 5). A statistical comparison of the two clusterings using CADM indicated that there was significant congruence between the clusterings (*P*<0.001), and that the extent of congruence was substantial (W metric=0.88, where the metric is 0 with no congruence and 1 with complete congruence). In both approaches, the diatom strains were separated into groups that generally correspond to diatom morphology. Raphid pennate diatoms (with the exception of the three noted above) formed a single cluster. Meanwhile several clusters of centric diatoms were formed, one main cluster containing most of the order Thalassiosirales (species in the genera *Thalassiosira* and *Skeletonema*), and some small clusters composed of polar centrics: one containing *Chaetoceros* species, and one containing the *D. brightwellii* strains.

**Fig. 6.**
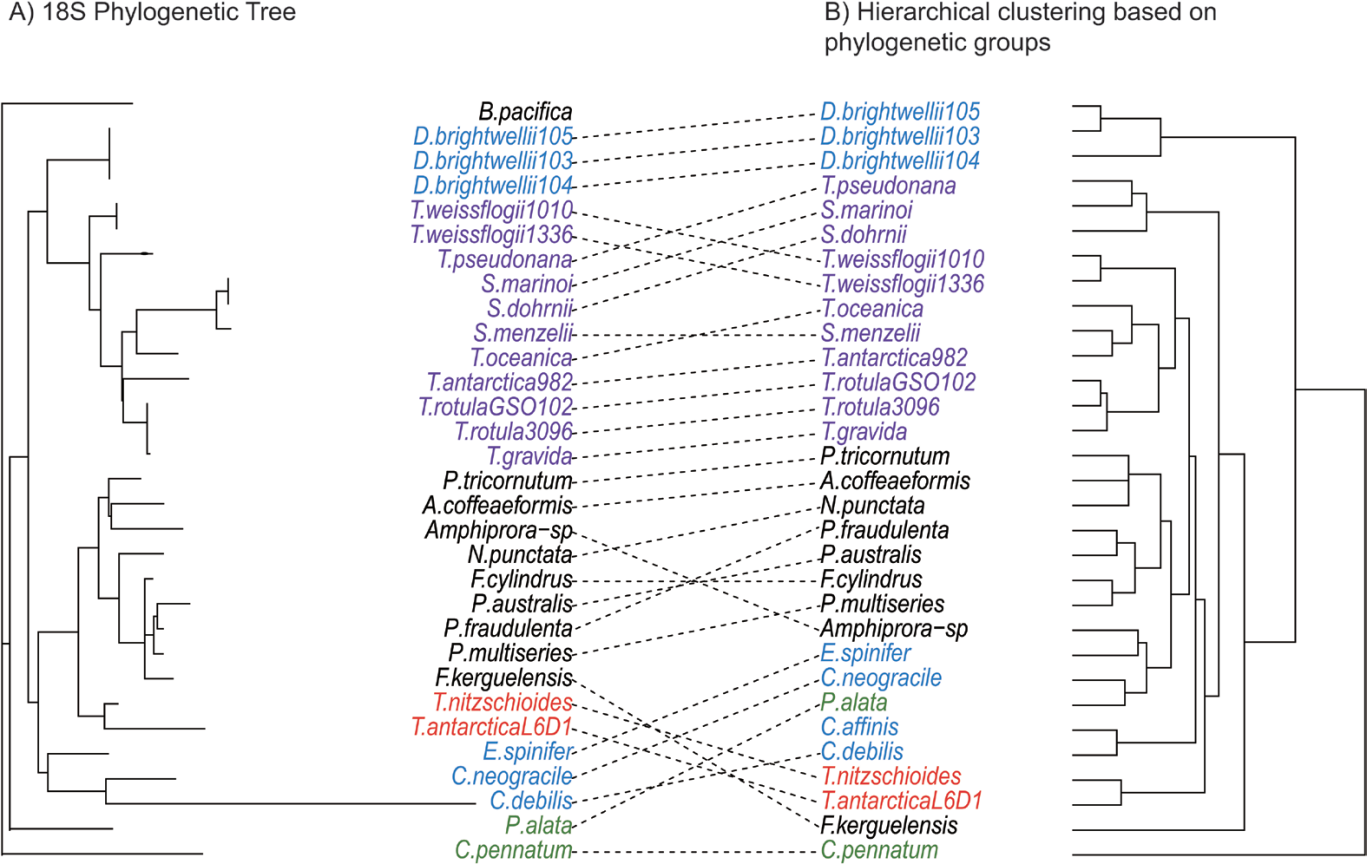
Comparison of diatom 18S rDNA phylogenetic tree and the dendrogram of CCM genes grouping by the protein phylogeny method. Black, raphid pennates; red, araphid pennates; blue, polar centrics; purple, radial centrics belonging to order Thalassiosirales; green, other radial centrics.

Additionally, a species phylogeny built from 18S rDNA sequences was substantially congruent with the clusterings based on genomic CCM gene content (Fig. 6; Supplementary Fig. S4). The CADM congruence test showed that the 18S phylogeny and CCM gene content clusterings had significant congruence (18S–phylogenetic groups, *P*<0.001; 18S–OrthoMCL, *P*<0.001), and the congruence was substantial in both cases (18S–phylogenetic groups, W=0.79; 18S–OrthoMCL, W=0.73). Most notably, the raphid pennates consistently formed a coherent cluster in both hierarchical clusterings and are a monophyletic clade. The polar centrics and Thalassiosirales, another monophyletic clade, were mostly contained within a single group in the hierarchical clusterings, but other radial centrics and araphid pennates were mixed into this cluster in both the OrthoMCL- and phylogentic-based clusterings. Furthermore, in the phylogenetic-based clustering, the *D. brightwelli* strains formed a distinct, distant cluster, incongruent with their phylogenetic position. The araphid pennates and radial centrics *C. pennatum* and *P. alata* did not show any clear correspondences between their positions in the species phylogeny and hierarchical clusterings.

## Discussion

CCMs have been studied extensively in cyanobacteria and the green alga *C. reinhardtii*, and have been reasonably well studied in the model diatoms *T. pseudonana* and *P. tricornutum* (Price *et al.*, 2008; Jungnick *et al.*, 2014; Hopkinson *et al.*, 2016). While the CCMs of different cyanobacterial species are generally similar, the CCM of *Chlamydomonas* differs greatly from that of the cyanobacteria, and there is almost no homology between the components of the systems, implying independent evolution rather than acquisition during the primary endosymbiotic event (Badger *et al.*, 1998, 2002; Raven *et al.*, 2011, 2012). Furthermore, the CCMs of *Chlamydomonas* and the model diatoms are quite different, with few components in common (Jungnick *et al.*, 2014), and even the CCMs of the two model diatoms, though making use of many similar components, are organized differently (Hopkinson *et al.*, 2016). Despite the paucity of well-characterized CCMs, the available evidence suggests that eukaryotic CCMs are subject to extensive diversification, leading us to explore the diversity of CCMs within diatoms, an ecologically important group of eukaryotic algae.

Newly available diatom transcriptomes together with the four sequenced genomes provide a broad sampling of marine diatom taxonomic and environmental diversity (Armbrust *et al.*, 2004; Bowler *et al.*, 2008; Keeling *et al.*, 2014). Most of the sequenced strains were isolated from the Pacific or Atlantic Ocean, but some strains such as *C. pennatum* L29A3, *F. kerguelensis* L2_C3, and *Thalassiothrix antarctica* L6_D1 were isolated from the Southern Ocean. The diatoms are disproportionately from coastal waters, but many came from open-ocean environments, and several were obtained from estuaries (e.g. *Skeletonema marinoi* SkelA, *Skeletonema dohrnii* SkelB, and *Thalassionema nitzschioides* L26_B). Our data set included the four major diatom morphologies, with 7 polar centrics, 13 radial centrics (11 belonging to the order Thalassiosirales), 9 raphid pennates, and 2 araphid pennates (Table 1).

The primary components of the diatom CCM that have been identified to date are SLC4 bicarbonate transporters and several groups of CAs, of which the α-, γ-, and δ-CA families are widespread among diatoms (Roberts *et al.*, 1997; Tachibana *et al.*, 2011; Nakajima *et al.*, 2013; Samukawa *et al.*, 2014). While the β-CAs from *P. tricornutum* and ζ-CAs from *T. weissflogii* are the best known and most well characterized CAs from diatoms (Tanaka *et al.*, 2005; Xu *et al.*, 2008), a BLAST search using members of these families failed to turn up homologs in more than a handful of the diatom strains examined. Consequently, further analysis focused on the four common gene families (SLC4s, α-, γ-, and δ-CAs). These CCM components were identified in the transcriptomes and genomes of diatoms using BLAST and HMM analyses. A limitation of the data set is the large number of transcriptomes since not all genes will necessarily be expressed under the culturing conditions used to generate the data. We compared the CCM components found in the *P. tricornutum* genome with those present in a *P. tricornutum* transcriptome (Levering *et al.*, 2017) and found that 16 of the 18 genes present in the genome were also expressed in the transcriptomes. This provides additional confidence that the CCM genes are generally expressed and that our analyses were not grossly biased. Further, poor quality transcriptomes (lack of completeness, bacterial contamination) were removed.

After compiling CCM protein sequences, sequences from each of the four CCM components (SLC4 bicarbonate transporters, α-CAs, γ-CAs, and δ-CAs) were grouped using OrthoMCL and phylogenetic trees into groups of related sequences that ideally play a similar functional role in the CCM (i.e. orthologous genes). In principle, the phylogenetic approach would be more appropriate for defining orthologs. However, generating robustly supported trees proved difficult with these sequences, and so a similarity-based method was used to complement the phylogenetic analysis. SLC4 bicarbonate transporters were grouped by both methods into a few major groups that contained sequences from most diatom strains (Figs 1–3), suggesting that nearly all diatoms have a similar set of conserved transporters that probably function in different roles in the cell. For example, one group may be localized to the plasma membrane bringing HCO_3_^−^ into the cell (Nakajima *et al.*, 2013), while another group may be embedded in the chloroplast membranes transporting HCO_3_^−^ into the chloroplast. In addition, SLC4 bicarbonate transporters from *T. pseudonana* and *P. tricornutum* were distributed throughout most of the major groups, which demonstrates that the CCMs of these model diatoms are in one respect representative of diatoms as a group.

In contrast to HCO_3_^−^ transporters, the α-CAs and δ-CAs only had one sequence group that contained sequences from most diatom strains (Figs 1, 2). Many of the remaining sequence groups showed specific taxonomic affiliations (Fig. 4; Supplementary Figs S1–S3). In some cases, these were broad taxonomic groups (e.g. pennates; Supplementary Fig. S1), but in other cases the groups were composed entirely of sequences from a single genus or even species. The most extreme examples of this are found in *D. brightwellii*, where both α-CAs and δ-CAs appear to have undergone extensive radiation (Fig. 4; Supplementary Figs S1, S2).

Overall, analysis of CA repertoires indicated that they are diverse and differentiated within diatoms, which in turn indicates that CAs have evolved rapidly within the diatom lineage, or perhaps that they have been acquired through horizontal gene transfer at different stages of diatom evolution. This perspective is also supported by experimental evidence, showing substantial differences among CA types, locations, and activities in *T. pseudonana* and *P. tricornutum* (Tachibana *et al.*, 2011; Samukawa *et al.*, 2014). For example, five α-CAs have been localized to the chloroplast membrane system in *P. tricornutum* and presumably function to control DIC flux into and out of the chloroplast, while in *T. pseudonana*, there is only one α-CA, which has been localized in the chloroplast stroma. δ-CAs have not been found in *P. tricornutum*, but four have been identified in *T. pseudonana* and are distributed throughout the cell (on the surface, in the mitochondria, and in the chloroplast membrane system) where they fulfill different roles (Tachibana *et al.*, 2011; Samukawa *et al.*, 2014).

In general, the CAs of these two model diatoms are of distinct subtypes and have divergent subcellular locations, which suggest that they perform different roles in the CCM in different diatoms. However, the γ-CAs are a notable exception. Two γ-CAs from *P. tricornutum* and three γ-CAs from *T. pseudonana* have been localized to the mitochondria (Tachibana *et al.*, 2011; Samukawa *et al.*, 2014). Mitochondrial localization of γ-CAs has also been found in the higher plant *Arabidopsis thaliana* (Parisi *et al.*, 2004), but CA activity has not been confirmed in these putative γ-CAs, suggesting that some γ-CAs might have different functions (Klodmann *et al.*, 2010). Phylogenetic analysis of diatom γ-CAs has shown that they are distant from those of *A. thaliana* (data not shown; Tachibana *et al.*, 2011). Within the diatoms, the three mitochondrial γ-CAs from *T. pseudonana* and one predicted mitochondrial γ-CA fell into four separate phylogenetically defined groups (Supplementary Fig. S3). Similarly, in OrthoMCL groupings, mitochondrial γ-CAs from both *T. pseudonana* and *P. tricornutum* fell into separate, conserved groups, suggesting that these conserved γ-CAs are mitochondrial in other diatom species. The consistent localization of multiple γ-CAs to the mitochondria in both diatoms and higher plants suggests that they play an important, functionally conserved role in these organisms. What that role is remains unclear, but notably a γ-CA subcomplex is found in the mitochondrial complex I of photoautotrophic eukaryotes, such as green algae and plants, but not in that of the heterotrophic eukaryotes such as fungi and mammals, suggesting that these γ-CAs are related to photosynthetic carbon metabolism (Hunte *et al.*, 2010; Klodmann *et al.*, 2010). Diatoms are unusual for algae in that they have a urea cycle (Armbrust *et al.*, 2004), and a possible role for the mitochondrial CAs is to generate bicarbonate for urea cycle metabolism, a known role for human mitochondrial CAs (van Karnebeek *et al.*, 2014).

Hierarchical clustering was used to assess relationships among diatoms based on their CCM gene content and then compared with a species phylogeny to assess the extent of vertical inheritance of diatom CCM components and repertoires. The CCM gene repertoire and 18S phylogeny are generally in agreement, and both show certain consistent correspondences between CCM genetic structure and diatom morphology: raphid pennates formed a coherent group and centric diatoms generally clustered together, but the radial centrics (*Proboscia alata* PI_D3) and araphid pennates (*Asterionellopsis glacialis* CCMP134, *Thalassiothrix Antarctica* L6_D1, and *Thalassionema nitzschioides* L26_B) were mixed in, apparently haphazardly, with the centrics cluster. Nonetheless, the CCM has developed substantially within diatom lineages and there is variation between the species phylogeny and clusterings based on CCM genetic structure. The most notable concordance was among the raphid pennates, which formed consistent groups in the CCM-based clustering and are a monophyletic taxon. The polar centrics and Thalassiosirales also showed substantial congruence between the species phylogeny and CCM gene clusterings, but there were some interesting disparities. *Ditylum brightwellii* belongs to order Lithodesmiales, which is a sister group of Thalassiosirales based on SSU rRNA (Medlin and Kaczmarska, 2004). However, the three *D. brightwellii* strains formed a distinct grouping based on CCM genetic structure, driven by diversification of CAs within this species (Figs 1, 2). The remaining radial centrics and araphid pennates were distributed throughout the CCM gene clusterings, with no obvious correspondence to their phylogenetic positions. In summary, clearly diatom CCMs are diverse and there is evidence both for gradual developments of CCMs during the evolution of diatom species and for anomalous CCM development in certain diatom taxa.

Insight into potential factors driving CCM diversification is found in the evolutionary history of diatoms. Fossil evidence indicates that eukaryotic marine algae originated ~1.6–1.8 Gya (Knoll *et al.*, 2006), and molecular phylogenetic analysis suggests that Rubisco evolved before the origin of oxygenic photosynthesis (Tabita *et al.*, 2007, 2008*a*, *b*). All oxygenic photoautotrophs that have evolved since that time use Rubisco for photosynthetic carbon fixation (Hohmann-Marriott and Blankenship, 2011). Diffusive supply of CO_2_ to Rubisco was presumably the ancestral mechanism of carbon supply in oxygenic photoautotrophs since CO_2_ concentrations were high and O_2_ was relatively absent early in earth’s history. However, as CO_2_ decreased and O_2_ increased, reliance on diffusive CO_2_ supply would begin to result in lower rates of fixation by Rubisco and increased rates of photorespiration. To overcome these inefficiencies, the CCM appeared as an important evolutionary response to maintain photosynthetic performance (Raven *et al.*, 2011, 2012). There may have been isolated niches where CO_2_ concentrations were low and hence CCMs were advantageous even when global CO_2_ concentrations were high, including biofilms, high pH regions, and endolithic habitats. CCMs may have evolved in these niches and then have expanded into the open ocean and other habitats as global CO_2_ concentrations declined.

The timing of atmospheric and oceanic oxygenation is still hotly debated (Lyons *et al.*, 2014) but, regardless, rising oxygen concentrations will drive down CO_2_ concentrations. Based on this, and the lack of similarity between the CCMs of green algae and cyanobacteria, CCMs of cyanobacteria and nascent green lineage algae most probably arose independently during the period of rising global oxygen prior to the Phanerozoic. Similarly, diatom CCMs share little similarity and the diatom lineage arose during the late Phanerozoic; therefore, we propose that the transport portion of diatom CCMs arose early in diatom evolution in the last 150 million years. Supporting this is the conserved presence of the SLC4 transporters, which appear to have originated from the exosymbiotic partner from which diatoms derived. In contrast, it seems likely that extreme variability in CO_2_ concentrations in the late Phanerozoic led to multiple ‘redesigns’ of the diatom CA repertoire and the specific tuning of Rubisco, such as the repeated glacial intervals of the Pleistocene 2.1 Mya (Raven *et al.*, 2011). Although there is no direct fossil evidence or molecular clocks to show the origin of CCMs, positive selection on form ID Rubisco in Bacillariophyta (diatoms) and Haptophyta during periods of low CO_2_ could relate to the origin or re-engineering of CCMs in these taxa (Young *et al.*, 2012). These repeated oscillations of periods of low and high CO_2_ could induce periodic pressure to develop or redesign the CCM, leading to continual diversification of the CCM, as observed in our analysis of diatom CCM gene content.

## Conclusions

Analyzing SLC4 bicarbonate transporters and CAs, the key known components of diatom CCMs, in the genomes and transcriptomes of 31 diatom strains revealed a great diversity of CCM architecture within diatoms. Much of this diversity corresponds to diatom species phylogeny, but the CCM has diverged substantially in some lineages. While SLC4 bicarbonate transporters are generally similar among diatom species, there has been extensive development of α- and δ-CAs within certain taxonomic groups. Comparable with this diversity among diatom CCMs, Young *et al.* (2016) have found extensive variation in diatom Rubisco kinetics, and in particular observed that some diatom Rubiscos had very high CO_2_ half-saturation constants, which would require a highly active CCM to saturate carbon fixation. They argued that diatom Rubisco traits suggested that individual diatom species trade-off investment in Rubisco content with resource allocation to the CCM. While the results of our study cannot specifically support this hypothesis, the extensive genetic diversity among diatom CCMs is consistent with the idea that resource allocation to the CCM differs greatly among diatom species.

## Supplementary data

Supplementary data are available at *JXB* online.

Fig. S1. Maximum parsimony phylogenetic tree of δ-CAs.

Fig. S2. Maximum parsimony phylogenetic tree of α-CAs.

Fig. S3. Maximum parsimony phylogenetic tree of γ-CAs.

Fig. S4. Comparison of 18S diatom species phylogeny and hierarchical clustering of diatom CCM species based on CCM gene content as determined using protein sequence similarity (OrthoMCL).

## Supplementary Material

supplementary_figures_S1_S4
