# Supplementary material for: The diversity of CO2-concentrating mechanisms in marine diatoms as inferred from their genetic content
Source: J Exp Bot. 2017 May 16;68(14):3937–48. doi: 10.1093/jxb/erx163 (PMC5853954; doi:10.1093/jxb/erx163)

Supplemental Material for:

The diversity of carbon dioxide concentrating mechanisms in marine diatoms as inferred from their genetic content

Chen Shen[1,2], Christopher L. Dupont[3], Brian M. Hopkinson[2]

[1]Qingdao Institute of BioEnergy and BioProcess Technology, Chinese Academy of Sciences, Qingdao, China

[2]Department of Marine Sciences, University of Georgia, Athens, GA, USA

[3]J. Craig Venter Institute, La Jolla, CA, USA

Supplemental Figure 1. Maximum parsimony tree of δ-CAs. Triangle symbols indicate bootstrap value greater than or equal to 50% and different colors indicate selected groups determined using the criteria described in the Materials and Methods.

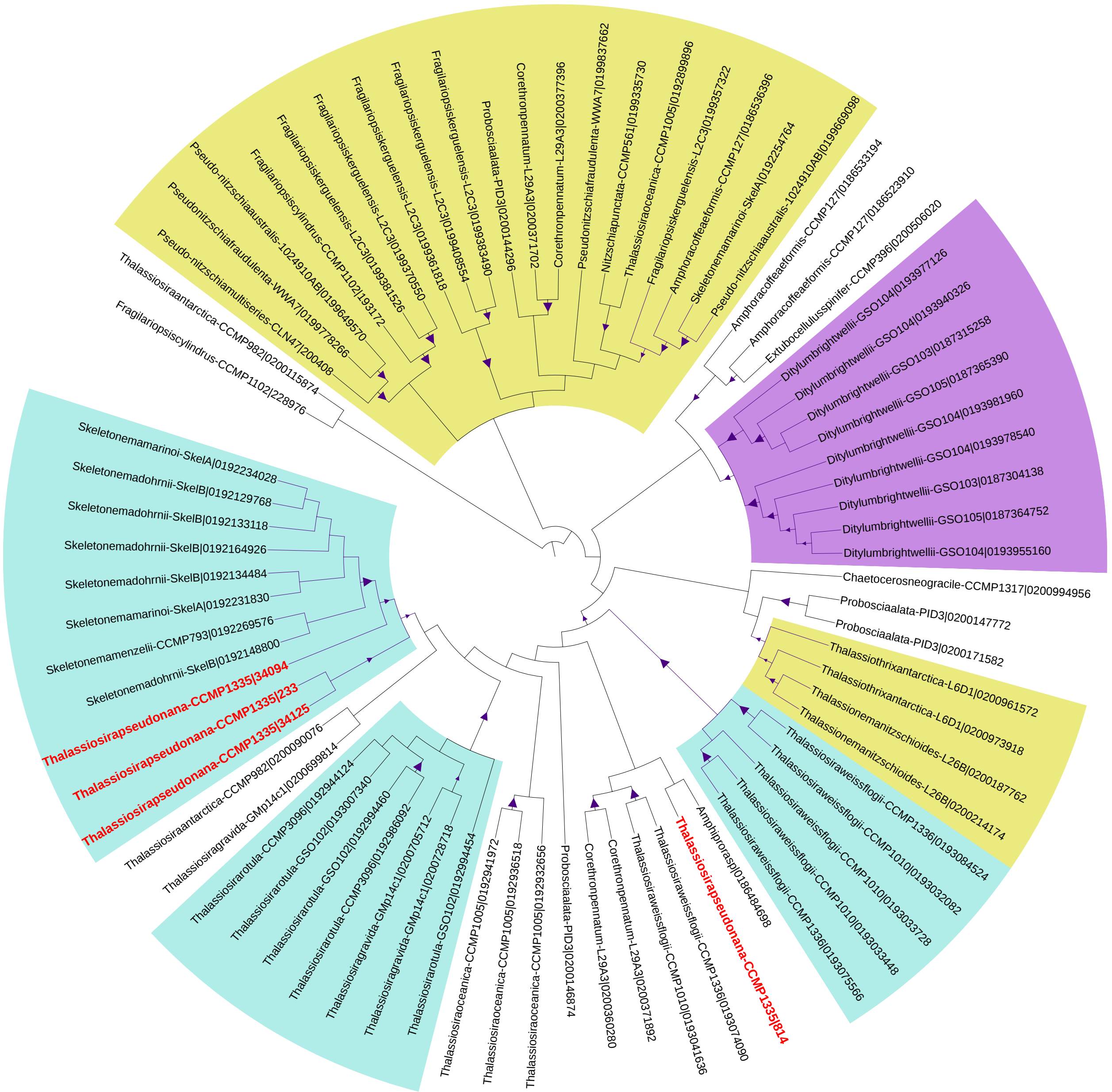

Supplemental Figure 2. Maximum parsimony tree of α-CAs. Triangle symbols indicate bootstrap value greater than or equal to 50% and different colors indicate selected groups determined using the criteria described in the Materials and Methods.

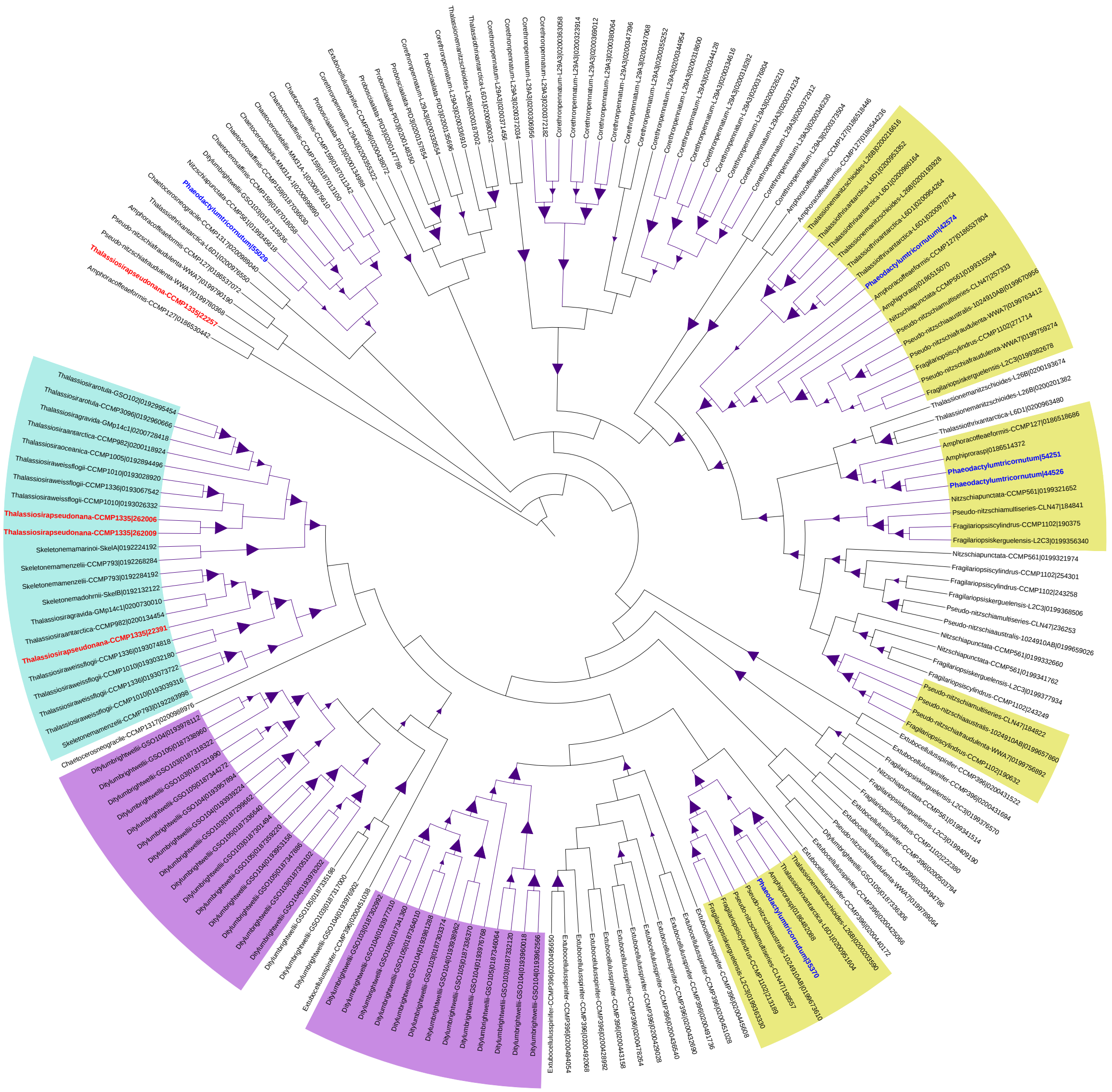

Supplemental Figure 3. Maximum parsimony tree of γ-CAs. Triangle symbols indicate bootstrap value greater than or equal to 50% and different colors indicate selected groups determined using the criteria described in the Materials and Methods.

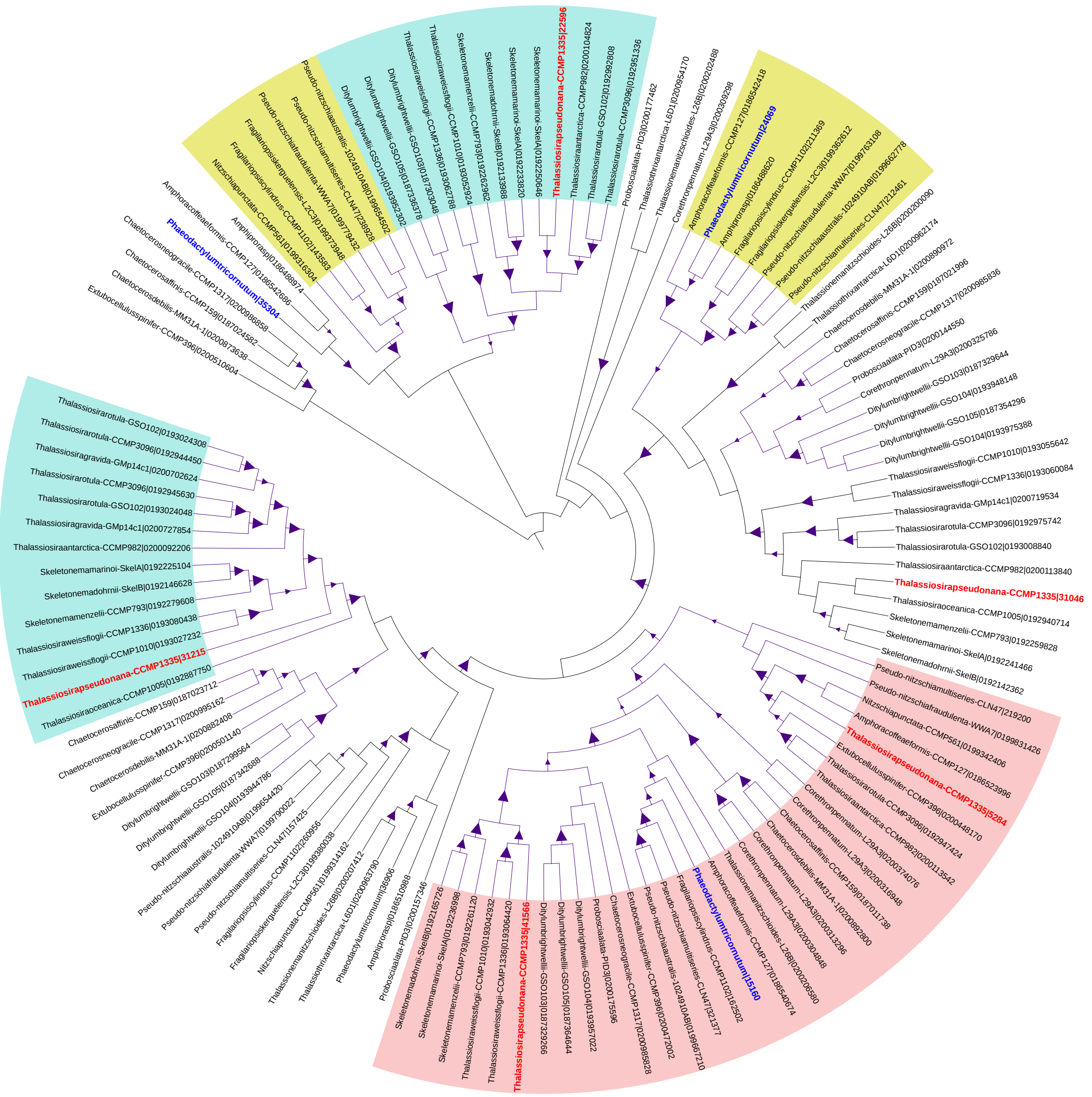

Supplemental Figure 4. Comparison of diatom 18S species phylogeny and species clustering based on CCM gene content defined using protein sequence similarity.

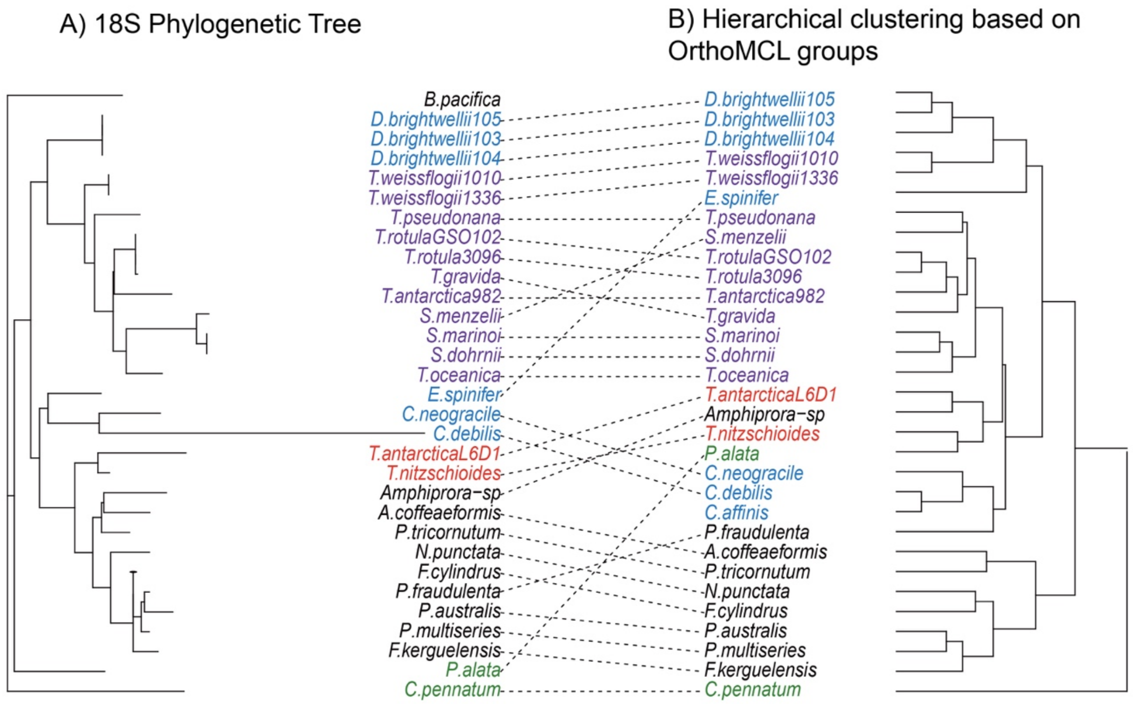

Supplement: supplementary_figures_S1_S4 [file erx163_suppl_supplementary_figures_s1_s4.pdf]
